# A retrospective view of pediatric cases infected with SARS-CoV-2 of a middle-sized city in mainland China

**DOI:** 10.1097/MD.0000000000023797

**Published:** 2020-12-18

**Authors:** Yanjun Kang, Zhong You, Kang Wang, Zijuan Dong, Jiajia Zhang, Yuanwang Qiu, Guizhi Ge

**Affiliations:** a Department of Pediatric Laboratory, The Affiliated Wuxi Children's Hospital of Nanjing Medical University; b Infectious Disease Department, The Fifth People's Hospital of Wuxi, Jiangsu Province; c Department of Infectious Disease, The Affiliated Wuxi Children's Hospital of Nanjing Medical University, Wuxi, China.

**Keywords:** children, COVID-19, SARS-CoV-2, Wuxi

## Abstract

The coronavirus disease 2019 (COVID-19) caused by SARS-CoV-2 had resulted in a global pandemic. A comprehensive analysis of pediatric COVID-19 cases is essential to decipher the natural features of children under the risk of this disease.

In the epidemic period, all the children infected with SARS-CoV-2 in Wuxi, a city with a stable medical system during the COVID-19 outbreak in China, were enrolled for comprehensive data documenting their clinical, prognosis, follow-up, treatment and various tests results. Combing their family cluster characteristics, the epidemiological, hospitalization, and transmission features of children with SARS-CoV-2 were analyzed and discussed.

A total of 7 children were enrolled, including 4 mild cases, 1 moderate case, and 2 asymptomatic cases. The common symptoms were fever and dry cough. The length of viral nucleic acid duration in nasopharynx varied and was irrelevant to the severity of the symptom, whether symptomatic or asymptomatic. Two cases showed viral nucleic acid positive recurrence after discharge from the hospital. A child with type 1 diabetes was also focused, for the elevated blood sugar during hospitalization. All these children had close contacts with their family members, some of those were confirmed COVID-19 cases.

We provided a holistic and detailed portrayal of the pediatric COVID-19 cases in a typical city of timely response to the epidemic. While the family cluster exhibits the major transmission mode, attention should be paid for the potential risk since the expanded social space of children in future.

## Abstract 2

Supplemental Digital Content is available in the text

## 1 Introduction

Coronaviruses, belonging to Coronaviridae, are a group of enveloped viruses with a positive single-strand RNA genome of 27-32 kb in size. Several species of the coronaviruses can infect humans and cause diseases ranging from mild to severe. Some coronaviruses, like 229E, NL63, OC43, and HKU1, are causative agents of the common cold. In contrast, the viruses cause severe illnesses like severe acute respiratory syndrome (SARS), and Middle East respiratory syndrome (MERS), are significant threats to human health.^[1]^ The coronavirus disease 2019 (COVID-19) is an emerging infectious disease caused by a novel coronavirus, SARS-CoV-2.^[2]^ This disease was firstly reported in Wuhan of Hubei province, China in December 2019, and had since spread worldwide, resulting in a global pandemic.^[3]^ To date, the confirmed cases in the world had overcome 10 million, of which more than 500 thousand people lost their lives.^[4]^

As an epidemiological study in China showed, 86.6% of cases were over 30 years old, and those under 20 in the infected crowd is 2.1%.^[5]^ Although children were not the research priorities since the COVID-19 epidemic began, there had been accumulating data documenting the pediatric cases.^[6–10]^ Generally, the children suffered milder disease burden compared to adults, while those under 5 and newborns were more likely to develop the severe form of this disease.^[7,11]^ Fever and cough are the most common signs or symptoms in pediatric cases as in symptomatic adults, but the alterations in radiological tests of children were fewer.^[12]^ Compared to the adults, the study on pediatric COVID-19 patients was neglected to some extent, due to the paucity of comprehensive and detailed view in the clinical, prognosis, follow-up, and family cluster features. As work resumption is taking place gradually in China and somewhere else at present, a retrospective study on multiple aspects of local pediatric COVID-19 cases is advantageous to coping the potential risk to children. Wuxi is a mid-sized city in the Yangtze River delta area of the Chinese mainland. Since the COVID-19 outbreak, a total of 55 diagnosed COVID-19 patients were reported, including 5 pediatric patients. Throughout the process, Wuxi did not greatly suffer from this epidemic, providing a typical research model as the city sustained a healthy medical system in this outbreak. In this study, we systematically summarize the hospitalization, follow-up, and family cluster features of the 5 reported pediatric cases together with 2 asymptomatic cases, which is beneficial to decipher the condition of children with timely treatment in a city with the normal medical system, further to guide the future coping strategy to this disease.

## 2 Methods

### 2.1 General research procedure

In this study, we included all the pediatric cases infected with SARS-CoV-2 in Wuxi, whether symptomatic or asymptomatic. At the beginning of the epidemic, the children who had been in fever clinics or close contact with COVID-19 patients in the local hospitals of Wuxi were focused. All these children were received real-time RT-PCR tests on nasopharyngeal swabs for SARS-CoV-2 by Wuxi Center for Disease Prevention and Control. The children with positive viral RNA result were regarded as a suspected COVID-19 patient and admitted to the designated hospitals, the Fifth People's Hospital of Wuxi and Wuxi Children's Hospital. During hospitalization, the clinical manifestation, laboratory tests results, real-time RT-PCR tests results, antiviral treatment process, prognosis condition were recorded. The cases were quarantined at home at least 2 weeks following the discharge from the hospital after 2 consecutive negative real-time RT–PCR tests. Then, the follow-up tests for SARS-CoV-2 nucleic acid were conducted every other week at least twice. In addition, the chest computed tomography (CT) was used to support the diagnosis when necessary.

### 2.2 Clinical types classification

According to the recommendations for the diagnosis, prevention, and control of the 2019 novel coronavirus infection in children (second edition), the severity types of COVID-19 consists of mild, moderate, severe, and critical. The mild type was only manifested as fever, cough, sore throat, nasal congestion, fatigue, headache, myalgia or discomfort, etc. There was no abnormality on chest CT imaging and no sepsis. The moderate type may have fever or no, accompanied by cough and other respiratory symptoms, and the CT examination suggested the change of viral pneumonia but did not reach the manifestation of severe pneumonia. On the basis of the clinical features, laboratory tests, and chest CT, the children infected with SARS-CoV-2 of this study were divided into the asymptomatic infection and some symptomatic types.

### 2.3 Clinical tests

The blood routine examinations, blood coagulation function, myocardial enzymes, procalcitonin, liver function, electrolytes, and chest computed tomography were conducted depending on the physical condition of the patients. The real-time RT-PCR tests on nasopharyngeal swabs were conducted using the protocol and test kits (BioGerm) recommended by the Chinese Center for Disease Control and Prevention. Two real-time RT-PCR tests were separated by at least 1 day.

### 2.4 Data collection and analysis

The general information of children and their family, obtained by interviews with their parents or other family members, along with clinical manifestation, various tests results, were recorded in special forms. Based on these data, we performed descriptive analyses such as proportions and frequencies for categoric variables.

## 3 Results

### 3.1 General information

A total of 7 children from 6 families who tested positive for SARS-CoV-2 were enrolled in this study, including 4 boys and 3 girls. Six children were Wuxi residents, and 1 was from Shanghai. Their age was ranging from 5 years to 15 years (mean age was 10.4 years). Except for 1 patient (Case5) diagnosed with type I diabetes for 3 years, the rest were healthy with no history of underlying diseases. All the children had been inoculated as planned by immunization programs of the Chinese government. The detailed information of each children can refer to the supplementary table.

Case1 and Case6 were from the same big family, they all returned from Xianning, an epidemic city of Hubei province. Case2 was infected by her mother. The Case3 had close contact with her mother, a COVID-19 patient confirmed in Anhui province. The Case4, together with his family member, once had family dinner with his relatives, of which one was confirmed case, and all his family members were infected. The Case5 was infected by his grandfather, a confirmed COVID-19 patient. The case7 was returned from Shanghai with her family, she and her mother were infected.

### 3.2 Clinical manifestation

**Figure 1:**
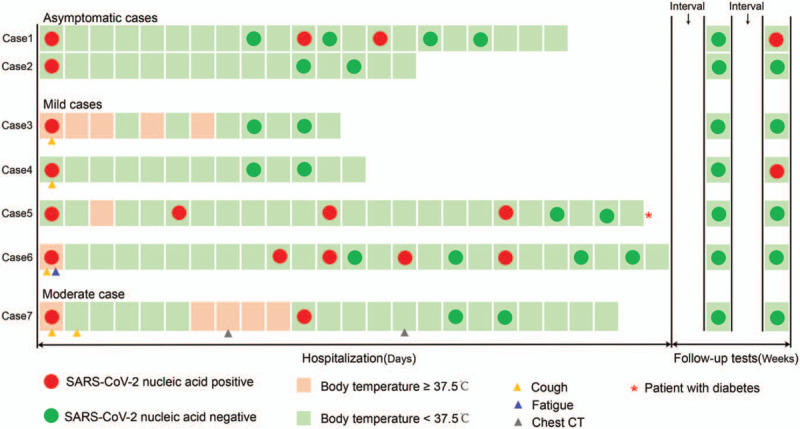
Schematic of the general clinical manifestation and RT-PCR tests results during hospitalization and follow-up.

Depending on the diagnosis guideline, of the 7 children there were: 2 asymptomatic cases, 4 mild cases, and moderate case, no severe or critical cases. The highest frequency of common symptoms of the 5 symptomatic children was mild fever (4 cases, 57%), no high fever cases. The case3 suffered fluctuant low fever (37.5–37.8°C) for 5 days in the first week of hospitalization. The case5 and case6 only had transient fever (37.5°C & 37.8°C) and recovered in the following day. For the moderate cases, the peak body temperature (38.7°C) was on admission, and recovered in the following day. But since the 7th day, a 4-day duration of milder fever (37.6–38.1°C) was present. The remaining major clinical symptoms were dry cough (4 cases, 57%), but not severe, which was relieved the next day. One case (case6) was with fatigue (14%) and soon relieved. Other symptoms like pharyngeal pain, dizziness, headache, vomiting, diarrhea, hemoptysis, and dyspnea were not observed. The general clinical process of the 7 cases was shown in Figure 1.

### 3.3 Laboratory tests and CT results

The routine blood tests were performed 2 or 3 times on each case, with an interval of 5 to 12 days (refer to supplementary table). About the leucocyte count, 1 decrease was observed in case5 on the 8th day of the disease course (3.73 × 10^9^/L), the remaining tests of this patient and others were fluctuating between 4.23–8.02 × 10^9^/L. The neutrophils ratio of 4 cases (case1, 2, 3, 5) was under the normal range slightly. And these cases were also shown higher lymphocytes ratio to a variable extent. Higher C-reactive protein was observed twice (8.7 and 12.2 mg/L) around the 9 to 16th day of the disease course in case6. For the liver function test, glutamic pyruvic transaminase in the serum was elevated up to 68 U/L in the second day of the disease course of case4 and decreased to normal range on the 7th day. Raised D-dimer was detected (0.4μg/ml) of case7 on admission. Hemoglobin, platelets, and procalcitonin of all cases were within the normal range. All the children showed negative of the viral RNA detection of influenza virus A and B. The test results of the myocardial enzyme, electrolyte, coagulation function, blood gas analysis, troponin, brain natriuretic peptide detection were within the normal range. The blood sugar of the case5 exceeded the normal range throughout the hospital stay, even though the hypoglycemic agent and special diet were applied. The average fasting blood glucose was 17.2, ranging 7.8 to 25.4. The routine blood test results of each children can refer to the supplementary table, and other tests results were not shown.

**Figure 2:**
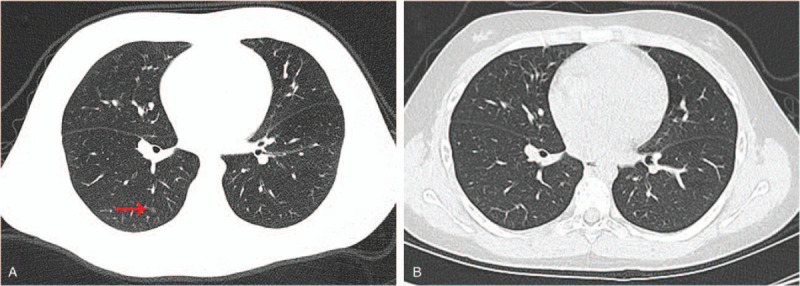
Chest CT results of the moderate case. The 2 results were separated by 7 days. The lesion in the lower lobe of the right lung was indicated by the red arrow.

The chest CT was conducted on the moderate case. The initial axial thin-section CT scan was acquired on the 8th day, a round nodular-like ground-glass opacification in the lower lobe of the right lung was observed. After 7 days, antiviral treatment was applied throughout the period, the opacification disappeared (Fig. 2).

### 3.4 Treatment

To relieve the symptoms, the 5 symptomatic cases were treated empirically with antiviral agents. Combination of 2 agents was administered, including interferon α spray (atomize or spray mouth, 7–10 days), lopinavir/ritonavir (oral administration, 2 times/d, 7 days). Besides, the moderate case was treated with the antibiotic amoxicillin clavulanate potassium. Hormone, intravenous gamma globulin, and mechanical ventilation therapy were not applied in the whole treatment process. No adverse reactions like nausea, diarrhea, elevated liver enzymes, bradycardia were observed.

### 3.5 Viral RNA tests, prognosis, and follow-up

All the children underwent SARS-CoV-2 nucleic acid test of nasopharyngeal swab during the stay in hospital irregularly as suggested by clinicians. The time for viral RNA tests results to turn negative was between 9 and 22 days, with an average of 16.6 days. The positive results were recurrent in the routine tests of 2 cases (case1, 6). Except for 1 patient with diabetes whose blood glucose was unstable, all the children discharged from hospital after confirmed negative results of viral RNA in nasopharynx 2 times. Nucleic acid tests of all cases were negative in the 2nd-week follow-up. However, as in the following 4th-week follow-up, 2 cases return positive (case1, 4) (Fig. 1). Then, the 2 children were subsequently admitted to the hospital for quarantine again. One week later, viral nucleic acid in their nasopharyngeal swab was confirmed negative 2 times (data not shown).

## 4 Discussion

As an emerging infectious disease, the COVID-19 has been causing tremendous destruction to the public health system and the social economy of the world. Wuxi, in Jiangsu province, like most cities outside Hubei province in China, suffered milder outbreak shock owing to a timely response to the situation. The SARS-CoV-2 monitoring was conducted targeting to the crowd including the people who had a trip to Wuhan or other epidemic areas, people had close contact with confirmed or suspicious infected person, and people had symptoms like fever, cough. All children in this study were under medical quarantine timely in the designated hospital as COVID-19 cases were confirmed among their family members. An overall and detailed process of their hospitalization and follow up were recorded and summarized in this study. This analysis of pediatric SARS-CoV-2 cases in Wuxi provides an insight into the clinical progress and coping strategy of this disease in the absence of vaccines and special drugs.

In our study, because of the limited cases, the pediatric patients did not show common clinical manifestation of diversity. Nonetheless, the fever and cough were still exhibited as the main clinical signs. Regarding the laboratory tests results, the subtle abnormalities of leucocyte count, neutrophils ratio, lymphocytes ratio, and C-reactive protein of some cases suggested the infection history and inflammatory responses. The tiny opacity under the CT image displaying potential lesion in the lung was rare in COVID-19 children just as other studies reports. It has become widely accepted that people of all ages were sensitive to SARS-CoV-2 but varied in the severity of clinical manifestation.^[5]^ Generally, children suffered milder disease, but those younger and newborns may develop into a severe illness.^[13]^ Lots of researches postulated that children were less susceptible to COVID-19, but this point is challenged.^[14,15]^ A retrospective cohort study, focused on symptomatic surveillance and contact tracing in Shenzhen, revealed that children had similar infection rate with the whole population (7.7% vs 6.6%), indicating children were as likely to be infected as adults.^[10]^

The pediatric patients with the underlying disease were not well documented owing to the limited cases. The child with type I diabetes in this study only showed slight fever, but the positive duration of SARS-CoV-2 nucleic acid is long (18 days). It is worth noting that, despite the hypoglycemic agents were applied, the blood glucose of this child was above the normal level continually through the hospitalization. In consideration of his parents were being quarantined, the child has poor treatment compliance and his routine diet was also interrupted. Then, whether the SARS-CoV-2 can impact the blood glucose of diabetes patients deserves further research. A study on the adult COVID-19 patients with diabetes shown a rapid deterioration of COVID-19 due to an inflammatory storm.^[16,17]^ Hence, the risk of the children with diabetes or other underlying disease should not be ignored as the vulnerable immune state.

The duration of the hospitalization among these children varied but did not correlate with the severity of symptoms. In other aspects, the persistently positive viral nucleic acid of some cases did not result in evident or severe clinical manifestation. The PCR positive results fluctuated in some cases, indicating the clearance of the virus in the body was unattainable for a short time. Strikingly, 2 cases, including an asymptomatic patient, the PCR tests were positive 3 weeks after discharge. Similar results had been revealed in adults.^[18]^ Even though some studies speculated that the false-negative PCR results or prolonged nucleic acid conversion may contribute to the “recurrence”, the prognosis of the disease deserves more efforts, which will help to make proper arrangement of the recovered cases.^[19]^

Consistent with other published results, the family cluster was the major epidemiological trait of the pediatric infected cases in this study. The reason, compared to adults, children have less social activity and a limited sphere of movement, but schools, other educational institutions, and childrens activity places were closed should also be considered. Asymptomatic transmission of SARS-CoV-2 is at the center of many discussions concerning it is easily overlooked.^[20]^ Although there is currently a large amount of data on children with COVID-19, there is no definitive evidence to elucidate the role of children in the transmission of the virus. Given that SARS-CoV-2 is likely to persist long in the population and the children could be potential facilitators of viral transmission, adequate preparation aiming at children activity place was essential in future widely open society.

## Acknowledgments

We thank the study participants, their family members, and all the clinical staff who participated in the fight against the COVID-19 epidemic at the Fifth People's Hospital of Wuxi and Wuxi Children's Hospital.

## Author contributions

**Conceptualization:** Yanjun Kang, Yuanwang Qiu, Guizhi Ge.

**Data curation:** Zhong You, Kang Wang, Zijuan Dong, Jiajia Zhang.

**Formal analysis:** Yanjun Kang, Guizhi Ge.

**Funding acquisition:** Yuanwang Qiu.

**Investigation:** Kang Wang, Jiajia Zhang.

**Methodology:** Yanjun Kang, Zhong You, Zijuan Dong, Jiajia Zhang, Yuanwang Qiu.

**Resources:** Yuanwang Qiu.

**Supervision:** Guizhi Ge.

**Writing – original draft:** Yanjun Kang, Yuanwang Qiu, Guizhi Ge.

**Writing – review & editing:** Yanjun Kang, Yuanwang Qiu, Guizhi Ge.

## Supplementary Material

Supplemental Digital Content
