# Supplementary material for: A retrospective view of pediatric cases infected with SARS-CoV-2 of a middle-sized city in mainland China
Source: Medicine (Baltimore). 2020 Dec 18;99(51):e23797. doi: 10.1097/MD.0000000000023797 (PMC7748305; doi:10.1097/MD.0000000000023797)
Supplement: Supplemental Digital Content [file medi-99-e23797-s001.docx]

|  |  | Case 1 | Case 2 | Case 3 | Case 4 | Case 5 | Case 6 | Case 7 |
| --- | --- | --- | --- | --- | --- | --- | --- | --- |
| Sex |  | Male | Female | Female | Male | Male | Male | Female |
| Age (years) |  | 14 | 6 | 10 | 8 | 5 | 15 | 10 |
| Trip to Hubei Province |  | Yes | No | No | No | No | Yes | No |
| Family members infected |  | Yes | Yes | Yes | Yes | Yes | Yes | Yes |
| Underlying disease |  | No | No | No | No | diabetes | No | No |
|  |  |  |  |  |  |  |  |  |
| Blood routine test results |  |  |  |  |  |  |  |  |
|  | Normal range |  |  |  |  |  |  |  |
| Leukocyte(10^9/L) | 4-10 | 5.39-6.62 | 5.6-6.76 | 6.94-7.23 | 4.48-4.53 | 3.73-5.42 | 6.6-8.02 | 4.23-5.82 |
| Neutrophil ratio(%) | 50-70 | 36.5-48.1 | 39.4-47.9 | 37-42 | 52.2-53.6 | 34.7-46 | 58.6-63.6 | 44.9-59.8 |
| Lymphocyte ratio(%) | 20-40 | 38.1-42.9 | 39.8-48.4 | 50-51.2 | 32.35.7 | 40.5-52.6 | 24.2-26.4 | 24.1-38.1 |
| Monocyte ratio(%) | 3-8 | 8.6-16 | 8．1-9.3 | 6.5-8.6 | 6.7-8.8 | 6.8-9 | 8.4-15.3 | 14.4-14.6 |
| Neutrophil count(10^9/L) | 2-7 | 1.97-3.19 | 2.66-2.68 | 2.68-2.9 | 2.34-2.43 | 1.49-1.93 | 3.87-5.1 | 1.9-3.48 |
| Lymphocyte count(10^9/L) | 0.8-4 | 2.31-2.52 | 2.23-3.27 | 3.4-3.7 | 1.45-1.6 | 1.7-2.85 | 1.6-2.12 | 1.4-1.61 |
| Monocyte count(10^9/L) | 0.1-0.8 | 0.57-0.86 | 0.52-0.55 | 0.45-0.62 | 0.3-0.4 | 0.27-0.38 | 0.67-1.01 | 0.61-0.85 |
| Red blood cell count(10^12/L) | 5.2-6.4 | 5.11-5.22 | 4.51-5.47 | 4.7-4.85 | 4.24-4.32 | 4.87-5.78 | 4.72-4.97 | 4.98-5.14 |
| Hemoglobin(g/L) | 180-190 | 143-148 | 136-147 | 142-147 | 121-122 | 125-149 | 141-148 | 134-138 |
| Hematocrit(%) | 38-50.8 | 42 | 0.44-36.6 | 41-43 | 34-35 | 37-43 | 40-43 | 0.4-0.43 |
| Platelet count(10^9/L) | 100-300 | 281-283 | 245-369 | 344-385 | 228-253 | 165-170 | 180-241 | 209-220 |
| Red blood cell distribution width(%) | 11-15 | 11.8-12.2 | 12.1-36.6 | 11.3-36.7 | 12-12.4 | 12.1-12.7 | 12-12.2 | 12.8-12.9 |
| Mean platelet volume(fL) | 9-13 | 11.5-12.1 | 9.3-12 | 10.3-10.6 | 11.1-11.2 | 10.3-11 | 9.9-11.9 | 11.9-12.1 |
| Platelet distribution width(fL) | 9-17 | 14.5-15.6 | 9.7-14.6 | 10.7-11.9 | 12.2-12.9 | 11.4-12.8 | 10.8-15.6 | 13.9-15.5 |
| Platelet hematocrit(%) | 0.11-0.28 | 33-34 | 0.29-34 | 36-40 | 26-28 | 17-19 | 21-24 | 0.25-0.26 |
| C-reactive protein | <0.5 | 0-1.8 | 0.5-3.3 | 0.5-3.3 | 2.2-3.3 | 2-6.4 | 3.4-12.2 | 1.22-5.26 |

Supplementary table: The general information and blood routine test results of the cases enrolled in this study
